# Giant Vulvar Condylomata: Two Cases and a Review of the Literature

**DOI:** 10.1155/2019/1470105

**Published:** 2019-05-16

**Authors:** Funda Gungor Ugurlucan, Cenk Yasa, Omer Demir, Ozlem Dural, Ekrem Yavuz, Suleyman Engin Akhan

**Affiliations:** ^1^Istanbul University Istanbul Faculty of Medicine, Department of Obstetrics and Gynecology, Turkey; ^2^Istanbul University Istanbul Faculty of Medicine, Department of Pathology, Turkey

## Abstract

**Introduction:**

Giant vulvar condyloma is usually associated with the HPV subtypes 6 and 11 and is characterized by excessive growth of verrucous lesions on the genitals and/or perianal region. It may be observed in sexually inactive as well as sexually active women. Immunosuppression plays an important role in the development of the disease.

**Patients and Methods:**

We report two cases of giant vulvar condyloma together with the review of the literature.

**Results:**

One case was a 21-year old sexually inactive woman with a history of Type 1 Diabetes. Second case was a 20-year-old sexually active woman with a rapidly progressing disease and cervical dysplasia. Both cases were operated; all the condylomatous structures were resected with preservation of the anatomy and clitoral innervation and blood flow. Skin and subcuticular dehiscence was the only complication encountered in the first case.

**Conclusion:**

Main treatment of giant vulvar condyloma is surgical resection with maintenance of the vulvar anatomy. Preservation of especially the clitoral innervation as much as possible is very important.

## 1. Introduction

Genital human papilloma virus (HPV) infections are transmitted primarily through sexual contact, with a lifetime risk of 50-80% [1]. The highest rate of genital HPV infection has been identified in adults between 18 and 28 years of age [2]. Vulvar condyloma is a sexually transmitted disease caused by Human Papillomavirus (HPV). Giant vulvar condyloma, also called Buschke-Löwenstein tumor, was first described by Abraham Buschke and Loewenstein Ludwig in 1925 [3]. Giant condyloma is usually associated with the HPV subtypes 6 and 11 and is characterized by excessive growth of verrucous lesions on the genitals and/or perianal region and is considered benign with high rate of recurrence and low risk of malignant transformation [4]. Host's immunity plays an important role in the development of the disease. Main risk factors for giant condylomas include smoking, having multiple sexual partners, chronic genital infections, poor hygiene, and immune deficiencies. Surgical excision is the treatment of choice in the management of giant condylomas.

Here we present two cases with giant vulvar condylomas and their management with a review of the literature.

## 2. Cases

### 2.1. Case 1

**Figure 1:**
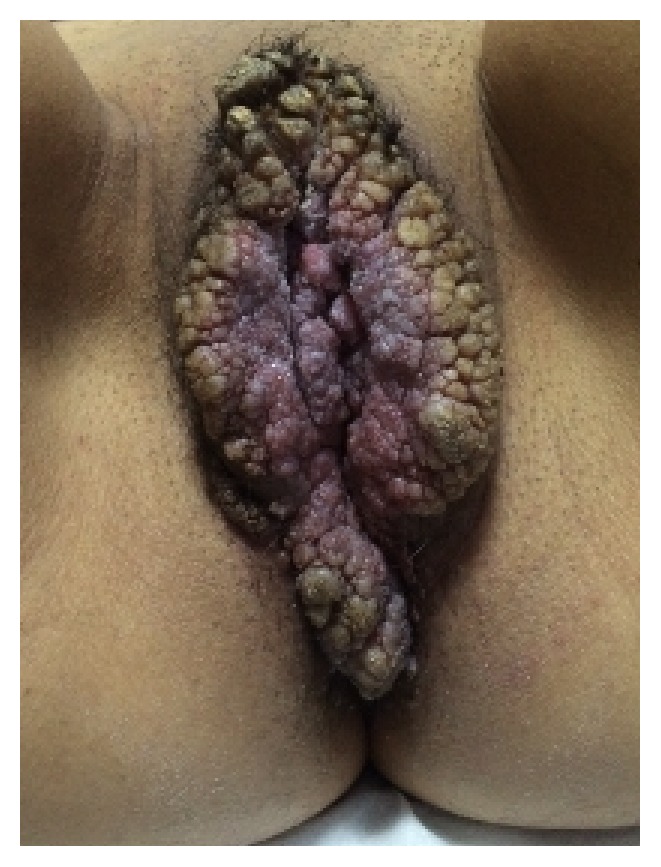
The appearance of Case 1 with distortion of labial and clitoral anatomy.

**Figure 2:**
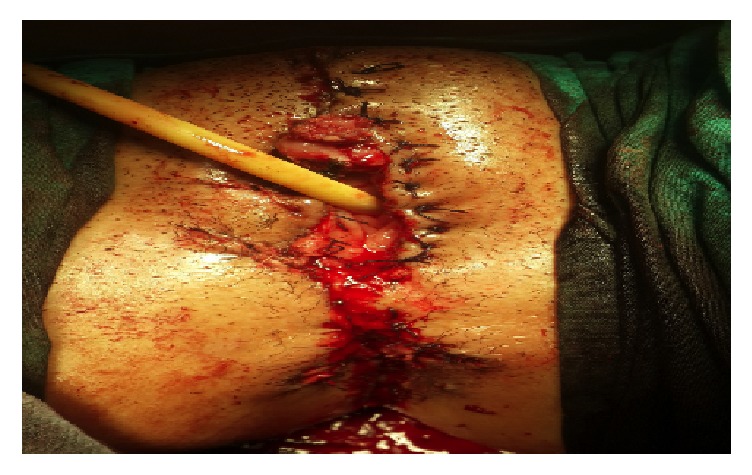
The appearance of Case 1 immediately after surgical resection.

**Figure 3:**
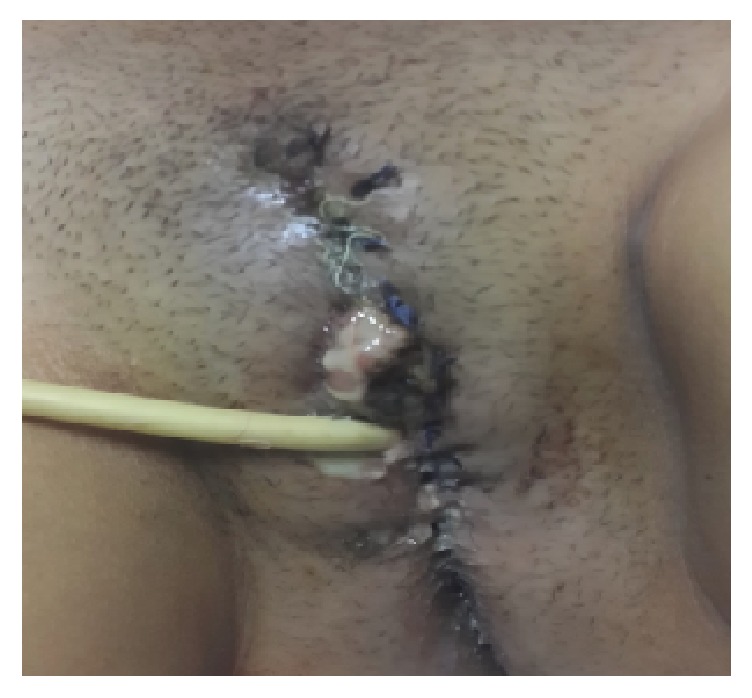
Postoperative appearance of Case 1.

**Figure 4:**
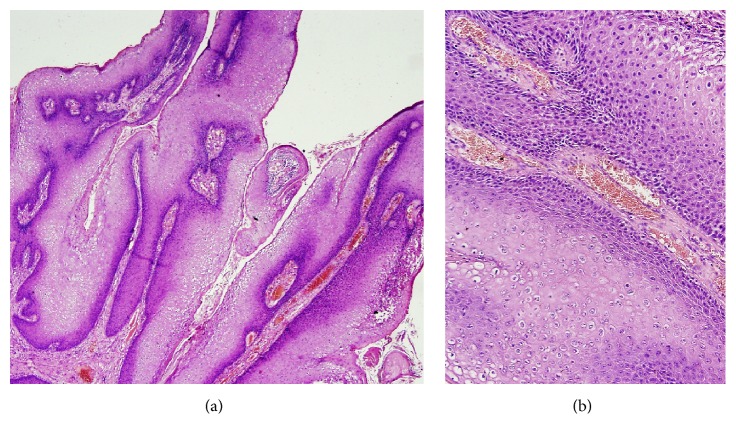
Histology of giant condyloma of Case 1. (a) x4 magnification (b) x20 magnification.

A 22-year-old sexually inactive woman presented with a rapidly growing mass in the vulva and perianal region. The lesions appeared 3 months before presentation and developed rapidly. The patient had no sexual activity. She was a regular smoker and the medical history included Type 1 diabetes mellitus for 15 years. The serologic screening for Hepatitis B (HBsAg), Hepatitis C (Anti-HCV), Human Immunodeficiency Virus (Anti-HIV), and syphilis (VDRL) was negative. Patient had no history of sexual diseases. On physical examination, a giant mass thought of as a giant condyloma, extending from the mons pubis till the anal mucosal lining and distorting the labial and clitoral anatomy was observed [Figure 1]. Biopsy of the lesion revealed a condyloma. Under general anesthesia a Foley catheter was introduced. Partial skinning vulvectomy was performed with preservation of clitoral and labial anatomy and all condylomatous structures were removed. Incisions were sutured primarily using single mattress sutures with No: 2/0 polyglactin [Figure 2]. No complications developed during the operation. Mobility was restricted and oral antibiotics were used in the postoperative period and the Foley catheter was left in situ. On the postoperative 5^th^ day, a 2-3 cm wound dehiscence involving the skin and subcuticular fatty tissue developed on the perineal area near the anal orifice and was treated with secondary healing using sitz baths, antibiotics, and topical creams [Figure 3]. Pathology report revealed condyloma acuminatum [Figures 4(a) and 4(b)]. No recurrences developed during one year of follow-up.

### 2.2. Case 2

**Figure 5:**
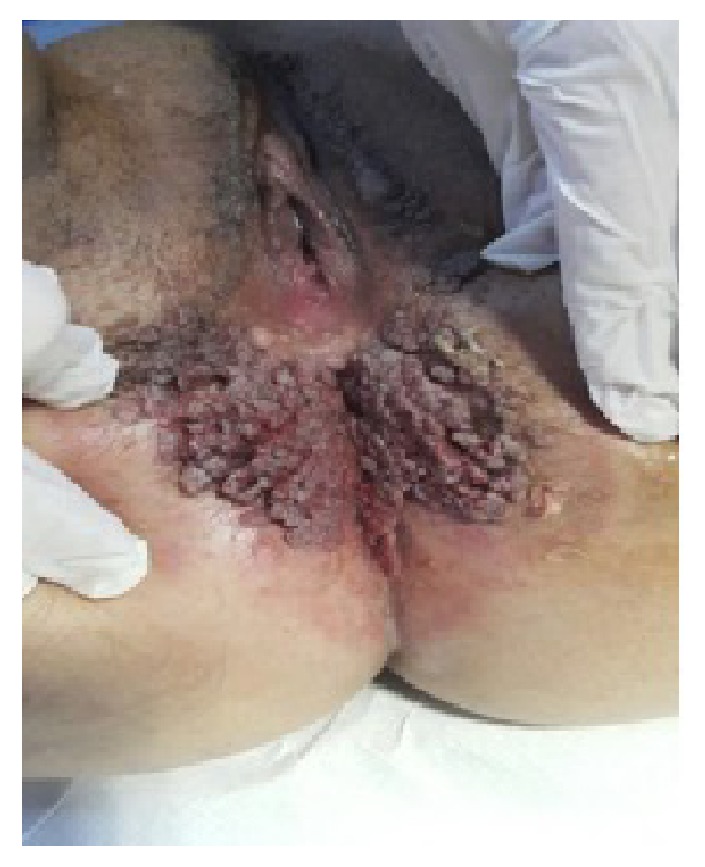
The appearance of Case 2 with main involvement of the anal region.

**Figure 6:**
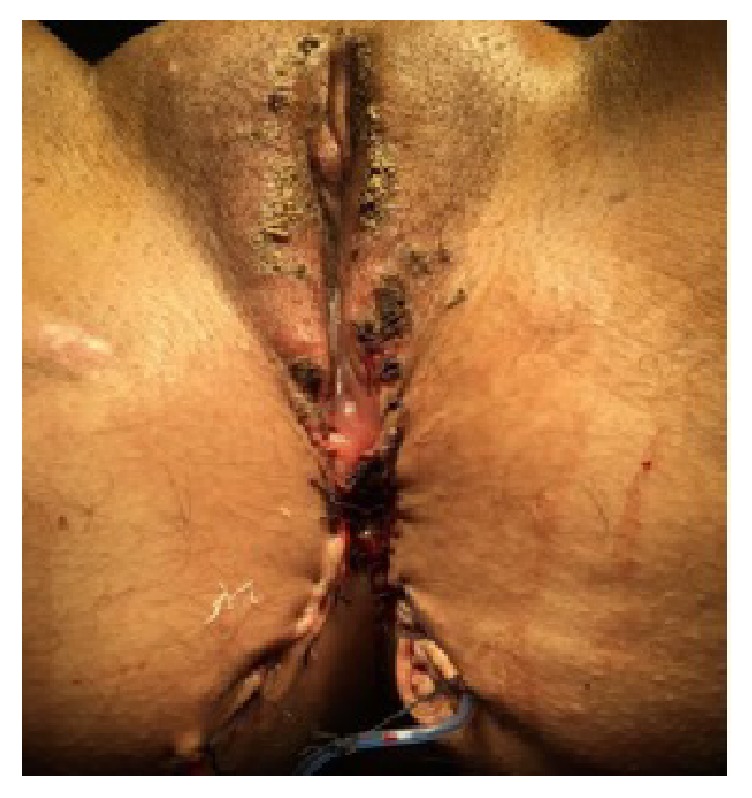
The appearance of Case 2 immediately after surgical resection.

**Figure 7:**
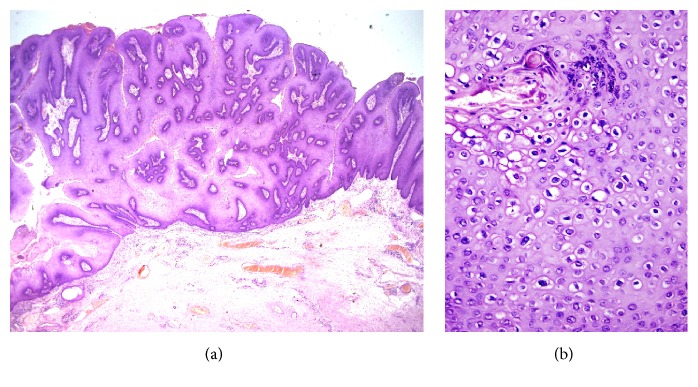
The histology of giant condyloma of Case 2. (a) x2 magnification (b) x40 magnification.

A 20-year old sexually active woman presented with a giant vulvar mass involving the vulva and the anal area. Lesions began to develop 5 months before presentation and developed rapidly. Medical history was unremarkable. Serologic screening for Hepatitis B (HBsAg), Hepatitis C (Anti-HCV), Human Immunodeficiency Virus (Anti-HIV), and syphilis (VDRL) was negative. Patient had no history of sexually transmitted diseases. Physical examination of the patient revealed condylomatous masses extending from the lower perineum to the intergluteal folds [Figure 5]. Biopsy of the lesion revealed a condyloma. PAP-smear was obtained and the result was low-grade squamous intraepithelial lesion. Colposcopy was performed and biopsy was taken from acetowhite areas which revealed cervical intraepithelial neoplasia (CIN)-II and follow-up was planned for cervical dysplasia. Under general anesthesia the condylomatous lesions were excised sharply and the condylomatous tissues around the clitoris were cauterized in order to preserve the anatomy and innervation of the clitoris. Drains were inserted in the gluteal incisions [Figure 6]. No complications developed during the postoperative period. Pathology report revealed condyloma acuminatum [Figures 7(a) and 7(b)]. No recurrences developed and the cervical dysplasia regressed during one year of follow-up.

## 3. Discussion

Buschke-Loewenstein tumor is a sexually transmitted disease caused by HPV subtypes 6 and 11, with benign histological features, but with excessive local growth and high recurrence rates [4]. They may rarely be associated with high-risk HPV types such as HPV type 16 [5]. Main site of appearance is the vulva in women and it is almost always preceded by condyloma acuminate [4]. The overgrowth results in hygienic problems, increases the risk of secondary infections, and distorts the genital image, which leads to social and psychological damage. Rarely giant condyloma may be associated with fistula formation. The host's immunodeficiency is important in the progression of disease; rapid progression and recurrences are usually associated with various types of immunodeficiency [6].

Rachman and Hasan reported a case of a 42-year-old woman presenting with giant vulvar condyloma diagnosed with systemic lupus erythematosus (SLE) one year ago and treated with surgical excision [7]. The patient was using methylprednisolone and mycophenolate mofetil and HPV types 6 and 11 were isolated from the vulva, vagina, and the cervix. Lube et al. reported that 289 of 5682 patients in the pediatric rheumatology unit were diagnosed with SLE and 4 of the 289 had giant condyloma acuminata (1.4%). All patients were under corticosteroid and immunosuppressant treatment [8].

CostaPinto et al. reported a 33-year-old patient diagnosed with SLE during pregnancy presenting with giant vulvar condyloma after delivery [9]. The patient was started on corticosteroids, immunosuppressant treatment including mycophenolate mofetil after pregnancy. Topical treatment with trichloroacetic acid, imiquimod, and podophyllin were only partially effective.

Petrini et al. reported a case of a 16-year-old patient with alcohol consumption and illicit drug usage [4]. The authors linked the appearance of the disease in this young patient with associated immunosuppression. Trombetta and Place reviewed the 51 cases published in the literature and observed that the disease was more frequent among men with a male to female ratio of 2.7 [10]. They also observed that the disease was becoming more common among younger people, with a rate of 3.5 in people less than 50 years of age. Both of our cases were young and one was sexually inactive. One had type I diabetes, which is an immunosuppressive state, and was a regular smoker. On the other hand, second case had no apparent immunosuppressive predisposing factor, but she was sexually active, yet the lesion developed quite rapidly.

Main treatment of giant vulvar condylomas is surgical excision. Apart from the aesthetic appearance of giant vulvar condylomas and the difficulty of surgical treatment, it is even more important to note that vulvar verrucous carcinoma should be included in the differential diagnosis of giant vulvar condylomas. Zerkan et al. reported a case of giant vulvar condyloma, which was resistant to conservative treatment and operated five times. The definitive pathologic examination revealed superficial vulvar carcinoma [11]. Liu et al. described six cases of vulvar verrucous carcinoma and three of these were accidentally diagnosed as giant vulvar condylomata [12]. Authors suggested that these tumors should be distinguished from giant condyloma acuminatum and well-differentiated squamous cell carcinoma, and surgery is the most effective treatment.

In conclusion, giant vulvar condylomas, which are caused by low-risk HPV types and treated with surgery, should be distinguished from verrucous carcinoma. Local aggressively growing vulvar condylomata, are seen more often in women with immunosuppression and should be treated as soon as they are diagnosed.
